# Cognitive trajectories of patients with focal ß-amyloid deposition

**DOI:** 10.1186/s13195-021-00787-7

**Published:** 2021-02-19

**Authors:** Si Eun Kim, Byungju Lee, Hyemin Jang, Juhee Chin, Ching Soong Khoo, Yeong Sim Choe, Ji Sun Kim, Sung Hoon Kang, Hang-Rai Kim, Song Hwangbo, Jee Hyang Jeong, Soo Jin Yoon, Kyung Won Park, Eun-Joo Kim, Bora Yoon, Jae-Won Jang, Jin Yong Hong, Duk L. Na, Sang Won Seo, Seong Hye Choi, Hee Jin Kim

**Affiliations:** 1 Department of Neurology, Samsung Medical Center, Sungkyunkwan University School of Medicine, Seoul, Korea; 2 Department of Neurology, Inje University College of Medicine, Haeundae Paik Hospital, Busan, Korea; 3 Department of Neurology, Yuseong Geriatric Rehabilitation Hospital, Pohang, Korea; 4 Samsung Alzheimer Research Center, Samsung Medical Center, Seoul, Korea; 5 Neuroscience Center, Samsung Medical Center, Seoul, Korea; 6 Neurology Unit, Department of Medicine, Universiti Kebangsaan Malaysia Medical Centre, Kuala Lumpur, Malaysia; 7 Department of Health Sciences and Technology, SAIHST, Sungkyunkwan University, Seoul, Korea; 8 Department of Neurology, Korea University Guro Hospital, Korea University College of Medicine, Seoul, Korea; 9 Department of Neurology, Ewha Womans University Seoul Hospital, Ewha Womans University School of Medicine, Seoul, Korea; 10 Department of Neurology, Eulji University Hospital, Eulji University School of Medicine, Daejeon, Korea; 11 Department of Neurology, Dong-A Medical Center, Dong-A University College of Medicine, Busan, Korea; 12 Department of Neurology, Pusan National University Hospital, Pusan National University School of Medicine and Medical Research Institute, Busan, Korea; 13 Department of Neurology, Konyang University College of Medicine, Daejeon, Korea; 14 Department of Neurology, Kangwon National University Hospital, Kangwon National University College of Medicine, Chuncheon, Korea; 15 Department of Neurology, Yonsei University Wonju College of Medicine, Wonju, Korea; 16 Department of Clinical Research Design and Evaluation, SAIHST, Sungkyunkwan University, Seoul, Korea; 17 Department of Digital Health, SAIHST, Sungkyunkwan University, Seoul, Korea; 18 Department of Neurology, Inha University School of Medicine, Incheon, Korea

**Keywords:** ß-Amyloid, ^18^F-flutemetamol PET, Longitudinal studies, Cognitive decline, Alzheimer’s disease

## Abstract

**Background:**

The presence of ß-amyloid (Aß) in the brain can be identified using amyloid PET. In clinical practice, the amyloid PET is interpreted based on dichotomous visual rating, which renders focal Aß accumulation be read as positive for Aß. However, the prognosis of patients with focal Aß deposition is not well established. Thus, we investigated cognitive trajectories of patients with focal Aß deposition.

**Methods:**

We followed up 240 participants (112 cognitively unimpaired [CU], 78 amnestic mild cognitive impairment [aMCI], and 50 Alzheimer’s disease (AD) dementia [ADD]) for 2 years from 9 referral centers in South Korea. Participants were assessed with neuropsychological tests and ^18^F-flutemetamol (FMM) positron emission tomography (PET). Ten regions (frontal, precuneus/posterior cingulate (PPC), lateral temporal, parietal, and striatum of each hemisphere) were visually examined in the FMM scan, and participants were divided into three groups: No-FMM, Focal-FMM (FMM uptake in 1–9 regions), and Diffuse-FMM. We used mixed-effects model to investigate the speed of cognitive decline in the Focal-FMM group according to the cognitive level, extent, and location of Aß involvement, in comparison with the No- or Diffuse-FMM group.

**Results:**

Forty-five of 240 (18.8%) individuals were categorized as Focal-FMM. The rate of cognitive decline in the Focal-FMM group was faster than the No-FMM group (especially in the CU and aMCI stage) and slower than the Diffuse-FMM group (in particular in the CU stage). Within the Focal-FMM group, participants with FMM uptake to a larger extent (7–9 regions) showed faster cognitive decline compared to those with uptake to a smaller extent (1–3 or 4–6 regions). The Focal-FMM group was found to have faster cognitive decline in comparison with the No-FMM when there was uptake in the PPC, striatum, and frontal cortex.

**Conclusions:**

When predicting cognitive decline of patients with focal Aß deposition, the patients’ cognitive level, extent, and location of the focal involvement are important.

**Supplementary Information:**

The online version contains supplementary material available at 10.1186/s13195-021-00787-7.

## Background

Accumulation of ß-amyloid (Aß) in Alzheimer’s disease (AD) can be detected via positron emission tomography (PET) imaging [1]. Although typical AD patients exhibit diffuse Aß deposition and the majority of cognitively normal individuals exhibit no Aß deposition in amyloid PET, a substantial portion shows focal Aß deposition (13.6% in cognitively unimpaired [CU], 16.0% in amnestic mild cognitive impairment [aMCI], and 26.7% in the AD dementia [ADD] stage) [2]. In clinical practice, amyloid PET is interpreted based on a dichotomous visual rating that regards focal Aß deposition as positive [3]. However, evidence supports the idea that focal Aß deposition is different from diffuse Aß deposition [2].

Managing patients with focal Aß deposition on PET scan is challenging. Will these patients face cognitive decline in the near future and if so, what initial features may predict cognitive decline? These questions remain to be unanswered as this subgroup has been rather neglected. In a previous cross-sectional study, we found that patients with focal Aß deposition involving larger regions or the striatum showed worse cognitive function [2]. Although there were studies that focused on regional Aß burden using standardized uptake value ratios (SUVR) [4–6], no previous study focused on longitudinal trajectories of focal Aß deposition based on visual rating.

Therefore, we used a visual rating of ^18^F-flutemetamol PET to assess Aß deposition and followed-up patients for 2 years with annual neuropsychological tests. We compared cognitive trajectories of patients with focal Aß deposition to those with no or diffuse Aß deposition. We hypothesized that cognitive decline would be influenced by baseline cognitive level, extent, and location of focal Aß deposition.

## Methods

### Participants

A total of 240 subjects were recruited in this study from June 2015 to January 2018. Of these recruited individuals, 112 were CU, 78 had aMCI, and 50 had ADD. The CU individuals were defined as normal performance adjusted for age and education according to the standardized cognitive tests [7, 8]. Patients fulfilling the following criteria as proposed by Petersen et al. were considered to have aMCI [9]: (1) self-reported memory impairment, (2) memory impairment on verbal or visual memory tests below − 1.5 standard deviation (SD) below mean, (3) normal daily functioning, and (4) non-demented. Patients with ADD met the core clinical criteria for probable ADD according to NIA-AA [10]. We enrolled all the participants from 9 referral centers in South Korea. Among them, 106 were recruited from the Samsung Medical Center and 134 from the Validation Cohort of Korean Brain Aging Study for the Early Diagnosis and Prediction of AD (KBASE-V). All participants underwent a thorough neurological examination, neuropsychological tests, brain MRI, and ^18^F-flutemetamol PET at baseline. Baseline blood tests were obtained including complete blood count, blood chemistry, vitamin B12 level, folate level, thyroid function, and syphilis serology to exclude other etiologies of cognitive decline. We excluded individuals with known neurological or psychological disorders such as central nervous system infection, epilepsy, and depressive disorders which might affect their cognitive performance. Patients with structural abnormalities detected from the MRI such as traumatic brain injury, brain tumors, or hydrocephalus were also excluded. Our study was approved by the Institutional Review Board to be conducted at all referral centers. Written informed consent from the participants and their caregivers were obtained prior to our study.

### ^18^F-flutemetamol PET acquisition and visual interpretation

^18^F-flutemetamol PET scans were performed in all 240 participants. As in our previous study, the instruments applied included Biography MCT PET/CT scanner (Siemens), Discovery STE PET/CT scanner (GE), Discovery 690 PET/CT scanner (GE), Discovery 600 PET/CT scanner (GE), and Gemini TF PET/CT scanner (Philips) [2]. After injecting 185 ± 10 MBq of ^18^F-flutemetamol intravenously followed by 10 mL of saline flush, all patients went through a 20-min PET scan (4 × 5 min dynamic frames). Low-dose computed tomography was conducted for attenuation correction prior to all scans. With the aid of 4 iterations and 16 subsets, the images were reconstructed using the Ordered Subsets Expectation Maximization Algorithm.

All ^18^F-flutemetamol PET images were independently reviewed by two board-certified neurologists, who were blinded to each patient’s clinical details. The readers were tasked with interpreting five brain regions (frontal, precuneus/posterior cingulate (PPC), lateral temporal, parietal lobes, and striatum) in each hemisphere after successfully completing the ^18^F-flutemetamol reader training provided by GE Healthcare [3, 11]. The region was considered positive when there was increased signal in cortical brain regions or striatum according to criteria defined by the manufacturer [11]. Inter-reader agreement of ^18^F-flutemetamol PET interpretation was excellent both for the binary classification at the regional level (kappa score = 0.94) and for the conventional final binary classification at the subject level (kappa score = 0.84). When there was disagreement between the readers, a consensus was reached during the re-read session.

We additionally performed semiquantitative analysis to calculate SUVR using the whole cerebellum as the reference region. There was a significant correlation between the number of ^18^F-flutemetamol uptake regions and global SUVR (Pearson’s correlation, *r* = 0.87, *p* <  0.001) (Additional file. Fig. S1).

The participants were then classified into three groups, namely the No-FMM (no ^18^F-flutemetamol uptake in any region), Focal-FMM (^18^F-flutemetamol uptake in 1–9 regions), and Diffuse-FMM (^18^F-flutemetamol uptake in all 10 regions) groups.

### Neuropsychological evaluation

The participants were evaluated using a standardized neuropsychological assessment: the Korean version of the Consortium to Establish a Registry for Alzheimer’s Disease Assessment Packet (CERAD-K) [12] or Seoul Neuropsychological Screening Battery (SNSB) [8], that included tests for four cognitive domains (frontal-executive, language, memory, and visuospatial functions).

The CERAD-K consisted of the Stroop Test (color reading) to evaluate frontal/executive function, the Korean version of the Boston Naming Test (K-BNT) to evaluate language function, 10-word list recall (20-min delayed recall) to evaluate verbal memory, constructional praxis (copying figures) to evaluate visuospatial function, and the Clinical Dementia Rating scale Sum of Boxes (CDR-SB) score to evaluate global cognitive function [12, 13]. Tests in SNSB included the Stroop Test (color reading) to evaluate frontal-executive function; the K-BNT to evaluate language function, Seoul Verbal Learning Test (20-min delayed recall) to evaluate verbal memory, Rey-Osterrieth Complex Figure Test (RCFT: copying) to evaluate visuospatial function; and the CDR-SB to evaluate global cognitive function [13, 14].

Annual neuropsychological tests were carried out by the qualified neuropsychologists. The norms of each test were based on 1987 normal Korean participants (for CERAD-K) or 1067 normal Korean participants (for SNSB). To analyze each cognitive domain, *z*-scores were used. These were based on the mean and SD of each measure in the age- and education-matched norms. We applied raw scores and controlled for age and education level when analyzing the CDR-SB.

### Statistical analysis

We performed variance analysis (ANOVA) for continuous variables and chi-square test for categorical variables, followed by the Bonferroni post hoc test to make a comparison of baseline demographic and clinical profiles among different diagnostic groups or stages. In order to evaluate the rate of cognitive decline in each subdomain (frontal-executive, language, memory, and visuospatial functions) in the Focal-FMM group in comparison to the No- or Diffuse-FMM groups, linear mixed-effects models were performed after including FMM groups (No-FMM, Focal-FMM, and Diffuse-FMM), time (quantified as years from baseline visit), and an FMM groups by time interaction as fixed effects. In the analysis of global cognitive function (CDR-SB), we further included baseline age and education as fixed effects. In all analyses, we included subjects as random effects.

To determine the rate of cognitive decline in the Focal-FMM group in comparison to the No- or Diffuse- FMM group in each cognitive level, we stratified patients into baseline cognitive level (CU, aMCI, and ADD) and performed aforementioned analyses for each cognitive level.

We further divided the Focal-FMM group according to the extent of FMM uptake: focal FMM uptake in 1–3 regions, 4–6 regions, and 7–9 regions. We performed the same analyses for each group.

In addition, we defined “regional Focal-FMM” as subjects with Focal-FMM that have ^18^F-flutemetamol uptake in that region irrespective of other regions. Subsequently, we compared the regional Focal-FMM group (frontal, PPC, lateral temporal, parietal lobes, and striatum) with the No- or Diffuse-FMM groups to investigate the cognitive function in relation to the focal FMM uptake regions. We corrected *P* values for multiple comparison (5 regions and 4 cognitive tests) using Bonferroni’s correction.

Med Calc Statistical Software version 19.3.1 (Ostend, Belgium) was applied for the ANOVA and chi-square tests; and SPSS software version 25.0 (SPSS, Inc.; Chicago, IL) was used for linear mixed-effects models.

## Results

### Demographic and clinical characteristics of the participants

All participants’ demographic and clinical profiles are summarized in Table 1. Among them, 18.8% (45/240) of patients were categorized into the Focal-FMM group. The Focal-FMM group was older and had more *APOE ε4* carriers than the No-FMM group. Also, differences in the distribution of cognitive levels were statistically significant across the groups. In the No-FMM group, the proportion of CU was high (68.0%); but in the Diffuse-FMM group, the proportion of aMCI (45.2%) and ADD (41.1%) were high. The number of ^18^F-flutemetamol uptake regions in each cognitive level is further provided in Additional file (Table S1).

**Table 1:** Demographic characteristics of participants

Uptake grade	No-FMM uptake (***n*** = 122)	Focal-FMM uptake (***n*** = 45)	Diffuse-FMM uptake (***n*** = 73)	***p*** No vs Focal uptake	***p*** No vs Diffuse uptake	***p*** Focal vs Diffuse uptake
**Age (Mean ± SD)**	69.2 ± 8.6	73.6 ± 8.0	71.1 ± 8.4	0.008	0.360	0.341
**Men (%)**	51 (41.8)	13 (28.9)	33 (45.2)	0.387	1.000	0.236
**Education, years (Mean ± SD)**	10.1 ± 5.0	10.3 ± 5.0	10.9 ± 4.5	1.000	0.822	1.000
***APOE ε4*** **carrier (*N*** **= 236)**	17/119 (14.3)	19/45 (42.2)	40/72 (55.6)	< 0.001	< 0.001	0.487
**Vascular risk factors**						
**Hypertension (%)**	59 (48.4)	19 (42.2)	26 (35.6)	1.000	0.250	1.000
**Diabetes (%)**	20 (16.4)	5 (11.1)	11 (15.1)	1.000	1.000	1.000
**Hyperlipidemia (%)**	45 (36.9)	14 (31.1)	10 (13.7)	1.000	0.002	0.069
**Diagnosis**				< 0.001	< 0.001	0.005
**CU (%)**	83 (68.0)	19 (42.2)	10 (13.7)			
**aMCI (%)**	34 (27.9)	11 (24.4)	33 (45.2)			
**ADD (%)**	5 (4.1)	15 (33.3)	30 (41.1)			

*P* values were corrected for multiple comparisons using the Bonferroni method

*FMM* ^18^F-flutemetamol, *APOE ε4* apolipoprotein ε4, *CU* cognitively unimpaired, *aMCI* amnestic mild cognitive impairment, *ADD* Alzheimer’s disease dementia

### Cognitive trajectories of patients with Focal-FMM uptake in each cognitive level

**Fig. 1:**
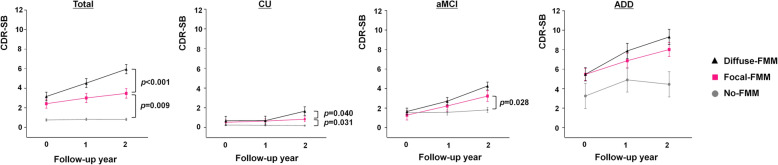
Cognitive trajectories of the Focal-FMM group in comparison to the No- or Diffuse-FMM group in each cognitive level. CU, cognitively unimpaired; aMCI, amnestic mild cognitive impairment; ADD, Alzheimer’s disease dementia; FMM, ^18^F-flutemetamol; CDR-SB, Clinical Dementia Rating Scale Sum of Boxes

Of the total participants, the Focal-FMM group showed faster cognitive decline than the No-FMM group (CDR-SB: 0.53 [0.20], *p* = 0.009) and slower cognitive decline than the Diffuse-FMM group (CDR-SB: − 0.84 [0.22], *p* <  0.001) (Table 2, Fig. 1).

**Table 2:** The rate of cognitive decline in the Focal-FMM uptake compared to the No- or Diffuse- FMM uptake in each cognitive level

	Total (***n*** = 240)		CU (***n*** = 112)		aMCI (***n*** = 78)		ADD (***n*** = 50)	
	Beta (SE)	***p***	Beta (SE)	***p***	Beta (SE)	***p***	Beta (SE)	***p***
Global cognition (CDR-SB*)								
Ref: No-FMM	0.53 (0.20)	**0.009**	0.22 (0.10)	**0.031**	0.83 (0.38)	**0.028**	0.36 (0.98)	0.716
Ref: Diffuse-FMM	− 0.84 (0.22)	**< 0.001**	− 0.32 (0.16)	**0.040**	− 0.26 (0.38)	0.505	− 0.86 (0.66)	0.200
**Cognitive subdomains**								
**Frontal-executive (Stroop test)**								
Ref: No-FMM	− 0.09 (0.09)	0.361	0.01 (0.10)	0.944	− 0.39 (0.19)	0.042	0.22 (0.36)	0.543
Ref: Diffuse-FMM	0.17 (0.11)	0.115	0.16 (0.15)	0.281	− 0.22 (0.19)	0.268	0.41 (0.26)	0.12
**Language (K-BNT)**								
Ref: No-FMM	− 0.12 (0.08)	0.133	− 0.08 (0.09)	0.385	− 0.23 (0.17)	0.177	− 0.01 (0.24)	0.976
Ref: Diffuse-FMM	0.15 (0.09)	0.088	− 0.15 (0.14)	0.263	0.02 (0.17)	0.915	0.24 (0.17)	0.169
**Memory (verbal memory)**								
Ref: No-FMM	− 0.15 (0.08)	0.064	− 0.07 (0.11)	0.544	− 0.32 (0.18)	0.078	− 0.14 (0.13)	0.288
Ref: Diffuse-FMM	0.11 (0.09)	0.244	0.53 (0.17)	**0.003†**	− 0.001 (0.19)	0.996	− 0.14 (0.09)	0.134
**Visuospatial (figure copying)**								
Ref: No-FMM	− 0.31 (0.15)	0.034	− 0.06 (0.12)	0.619	− 0.82 (0.37)	0.033	0.02 (0.56)	0.968
Ref: Diffuse-FMM	− 0.17 (0.16)	0.296	0.22 (0.19)	0.236	− 0.69 (0.38)	0.079	0.03 (0.39)	0.947

*FMM* ^18^F-flutemetamol, *SE* standard error, *CU* cognitively unimpaired, *aMCI* amnestic mild cognitive impairment, *ADD* Alzheimer’s disease dementia, *K-BNT* Korean version-Boston Naming Test, *CDR-SB* Clinical Dementia Rating Scale Sum of Boxes

*Values were adjusted for age and education

†*p* < 0.05 after Bonferroni correction for 4 tests (4 cognitive subdomains)

In the CU participants, cognitive decline in the Focal-FMM group was slower than the Diffuse-FMM group (CDR-SB: − 0.32 [0.16], *p* = 0.04; verbal memory: 0.53 [0.17], *p* = 0.003). Also, the Focal-FMM group showed faster cognitive decline when compared to the No-FMM group (CDR-SB: 0.22 [0.10], *p* = 0.031) (Table 2, Fig. 1).

In patients with aMCI, cognitive decline of the Focal-FMM group was faster than the No-FMM group (CDR-SB: 0.83 [0.38], *p* = 0.028); however, there was no significant difference compared to the Diffuse-FMM group (Table 2, Fig. 1).

In ADD patients, the rate of cognitive decline in the Focal-FMM group was not significantly different than the No- or Diffuse-FMM groups (Table 2, Fig. 1).

### Cognitive trajectories of patients with Focal-FMM uptake according to uptake extent

**Fig. 2:**
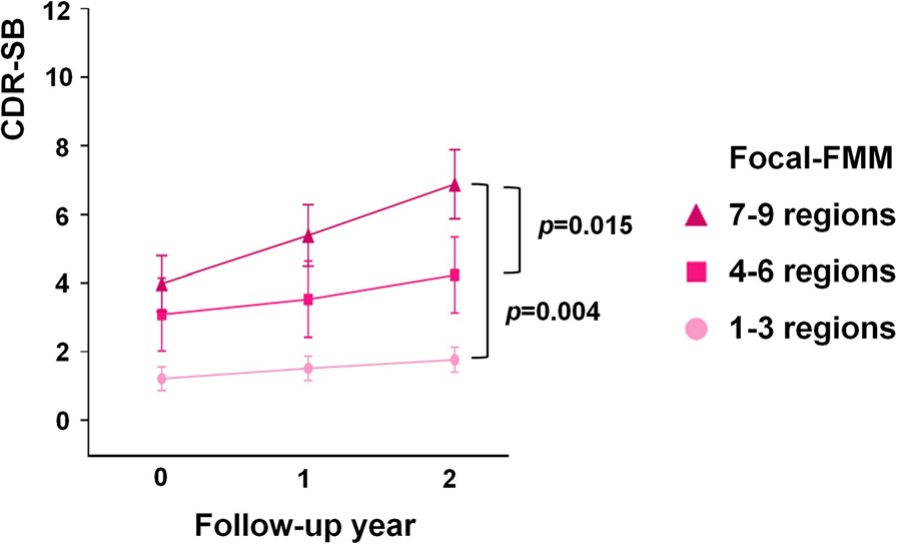
Cognitive trajectories of the Focal-FMM group according to the number of focal uptake regions. FMM, ^18^F-flutemetamol; CDR-SB, Clinical Dementia Rating Scale Sum of Boxes

We further divided the Focal-FMM group into 3 groups (1–3, 4–6 and 7–9 regions involved). Focal-FMM group involving 7–9 regions exhibited faster decline in CDR-SB compared to the Focal-FMM group with 1–3 or 4–6 regions involved (β[SE]: 1.13 [0.37], *p* = 0.004 (1–3 regions), β[SE]: 1.00 [0.40], *p* = 0.015 (4–6 regions)) (Fig. 2). The results remained to be significant after being adjusted for the diagnosis (β[SE]: 1.12 [0.37], *p* = 0.003 (1–3 regions), β[SE]: 0.97 [0.39], *p* = 0.016 (4–6 regions)). There was no significant difference in the rate of cognitive decline between Focal-FMM with 1–3 regions involved and Focal-FMM with 4–6 regions involved. We further performed linear correlation analysis using the number of FMM uptake regions as continuous variable. Within the Focal-FMM group, an increasing number of FMM uptake regions at baseline correlated with faster cognitive decline in CDR-SB (β[SE]: 0.18 [0.06], *p* = 0.002).

### Cognitive trajectories of patients with Focal-FMM uptake in each region

**Fig. 3:**
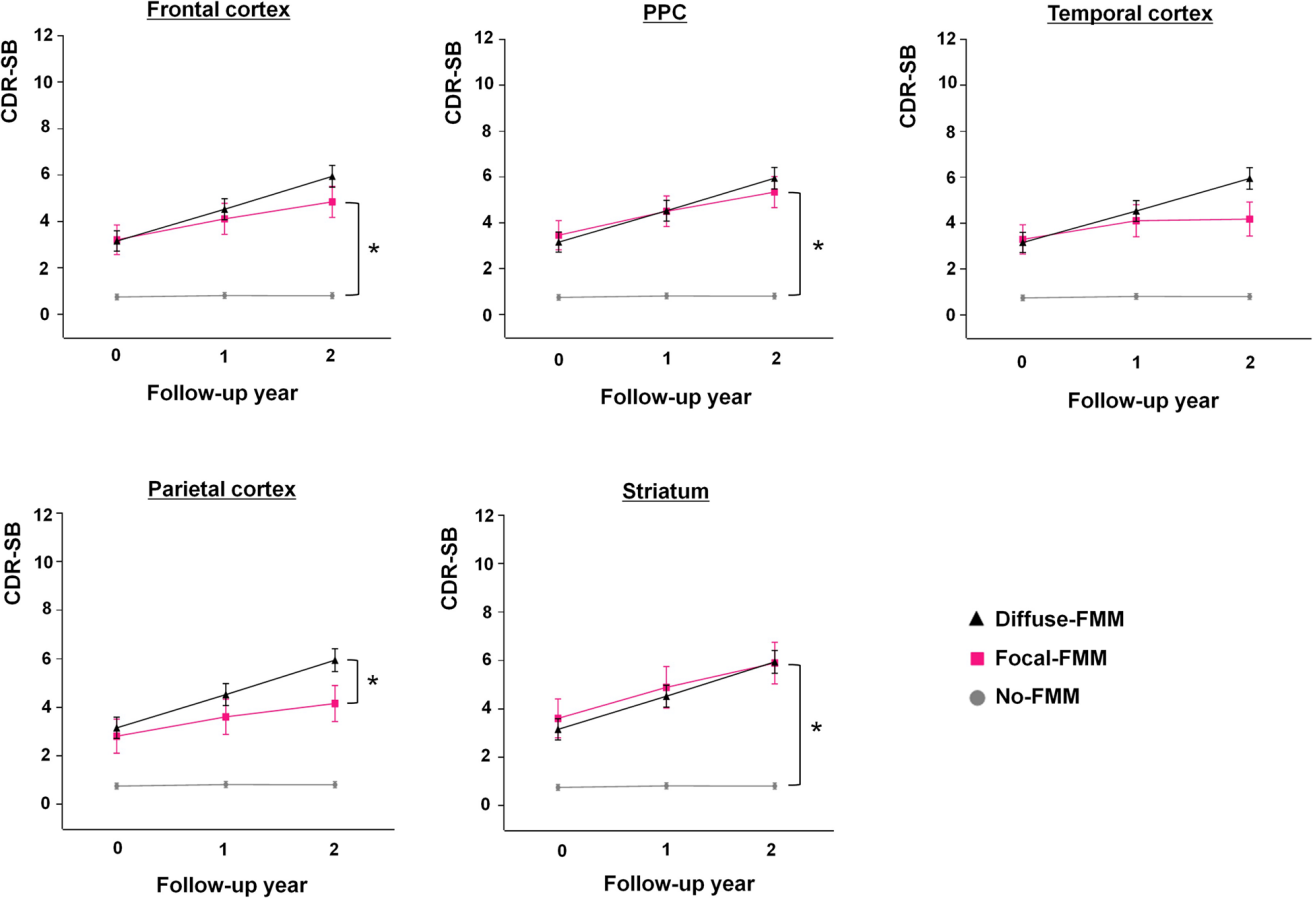
Cognitive trajectories of Focal-FMM patients in each region. FMM, ^18^F-flutemetamol; PPC, precuneus/posterior cingulate; CDR-SB, Clinical Dementia Rating Scale Sum of Boxes. *p < 0.05 after Bonferroni correction for 5 tests (5 regions)

In order to investigate the locational effects of Focal-FMM uptake on cognitive impairment, each regional Focal-FMM group was compared with the No- or Diffuse-FMM groups. The Focal-FMM group with frontal (CDR-SB: 0.74 [0.25], *p* = 0.003), PPC (CDR-SB: 0.85 [0.25], *p* = 0.001; verbal memory: − 0.33 [0.10], *p* = 0.001), and striatal (CDR-SB: 0.94 [0.29], *p* = 0.001) involvement showed faster cognitive decline compared to the No-FMM group after Bonferroni correction for multiple comparisons. No significant difference was found when compared to the Diffuse-FMM group (Table 3, Fig. 3). However, Focal-FMM with parietal involvement showed slower cognitive decline compared to Diffuse-FMM (CDR-SB: − 0.73 [0.27], *p* = 0.008) but no significant difference was found compared to the No-FMM group after Bonferroni correction for multiple comparisons (Table 3, Fig. 3). Focal-FMM with temporal involvement showed no difference in the rate of cognitive decline compared to the Diffuse-FMM or No-FMM groups.

**Table 3:** The rate of cognitive decline in Focal-FMM compared to No- or Diffuse-FMM uptake in each location of FMM uptake

	Frontal (***n*** = 29)		PPC (***n*** = 28)		Temporal (***n*** = 14)		Parietal (***n*** = 26)		Striatum (***n*** = 21)	
	Beta (SE)	***p***	beta (SE)	***p***	Beta (SE)	***p***	Beta (SE)	***p***	Beta (SE)	***p***
Global cognition (CDR-SB*)										
Ref: No-FMM	0.74 (0.25)	**0.003†**	0.85 (0.25)	**0.001†**	0.44 (0.36)	0.224	0.64 (0.25)	0.012	0.94 (0.29)	**0.001†**
Ref: Diffuse-FMM	− 0.63 (0.27)	0.020	− 0.51 (0.27)	0.060	− 0.92 (0.37)	0.014	− 0.73 (0.27)	**0.008†**	− 0.43 (0.31)	0.166
**Cognitive subdomains**										
**Frontal-executive (Stroop test)**										
Ref: No-FMM	− 0.11 (0.12)	0.352	− 0.18 (0.12)	0.142	− 0.17 (0.18)	0.351	− 0.08 (0.12)	0.495	− 0.11 (0.14)	0.423
Ref: Diffuse-FMM	0.14 (0.13)	0.277	0.07 (0.13)	0.582	0.09 (0.19)	0.641	0.17 (0.13)	0.201	0.14 (0.15)	0.337
**Language (K-BNT)**										
Ref: No-FMM	− 0.13 (0.10)	0.185	− 0.23 (0.10)	0.021	− 0.20 (0.14)	0.165	− 0.15 (0.10)	0.136	− 0.15 (0.11)	0.19
Ref: Diffuse-FMM	0.14 (0.11)	0.192	0.04 (0.11)	0.735	0.07 (0.15)	0.658	0.12 (0.11)	0.278	0.12 (0.12)	0.319
**Memory (verbal memory)**										
Ref: No-FMM	− 0.23 (0.10)	0.022	− 0.33 (0.10)	**0.001††**	− 0.21 (0.15)	0.165	− 0.08 (0.10)	0.425	− 0.23 (0.12)	0.048
Ref: Diffuse-FMM	0.03 (0.11)	0.767	− 0.07 (0.11)	0.492	0.05 (0.15)	0.745	0.18 (0.11)	0.113	0.03 (0.12)	0.824
**Visuospatial (figure copying)**										
Ref: No-FMM	− 0.37 (0.18)	0.045	− 0.52 (0.19)	0.007	− 0.06 (0.28)	0.836	− 0.56 (0.19)	0.004	− 0.49 (0.21)	0.022
Ref: Diffuse-FMM	− 0.23 (0.20)	0.246	− 0.38 (0.21)	0.066	0.08 (0.29)	0.789	− 0.42 (0.21)	0.044	− 0.35 (0.23)	0.119

*FMM* ^18^F-flutemetamol, *PPC* precuneus/posterior cingulate, *SE* standard error, *K-BNT* Korean version-Boston Naming Test, *CDR-SB* Clinical Dementia Rating Scale Sum of Boxes

*Values were adjusted for age and education

**†***p* < 0.05 after Bonferroni correction for 5 tests (5 regions)

**††***p* < 0.05 after Bonferroni correction for 20 tests (5 regions and 4 cognitive subdomains)

## Discussion

In the current study, we investigated the cognitive trajectories of patients with Focal-FMM uptake compared to those with No- or Diffuse-FMM uptake. The Focal-FMM group in the CU stage demonstrated slower cognitive decline than the Diffuse-FMM group and faster cognitive decline than the No-FMM group. The Focal-FMM group in the aMCI stage showed faster cognitive decline than the No-FMM group. In the Focal-FMM group, patients with a larger extent of Aß deposition showed faster cognitive decline. We also found that those with focal Aß deposition in the PPC, striatum, or frontal lobes showed faster cognitive decline than those in the No-FMM group. Taken together, cognitive trajectories of patients with focal Aß deposition differed from no- or diffuse Aß deposition according to the patients’ cognitive level, the extent, and the location of focal involvement.

We found that the Focal-FMM group showed different rates of cognitive decline according to the cognitive level at baseline. In the CU and aMCI stage, the rate of cognitive decline in the Focal-FMM group was faster than the No-FMM. Previous studies also reported that greater Aß deposition in CU was associated with faster cognitive decline [15–17]. It is well established that in the aMCI stage Aß positive patients show faster cognitive decline and are at higher risk of dementia progression (hazard ratio = 3.74) than Aß negative aMCI [18]. Our result further strengthens that CU or aMCI patients with even focal Aß deposition also show faster cognitive decline than Aß negative patients. Compared to the Diffuse-FMM group, the Focal-FMM group in the CU stage showed slower cognitive decline while in the aMCI stage there was no significant difference. It is possible that when Aß deposition is focal in the CU stage, it might be that they are at a very early stage in the AD process in which tau accumulation and neurodegeneration has not yet started or has just begun. In addition, in CU participants, the Focal-FMM group showed slower memory decline than the Diffuse-FMM group, which might be also explained by learning effect [19, 20]. A longer follow-up study is needed to track their cognitive trajectories.

We also found that the rate of cognitive decline in patients with focal Aß deposition was different according to the extent of focal FMM uptake. In a cross-sectional study, we previously showed that in the Focal-FMM group, cognitive scores reduced with increasing number of FMM uptake regions [2]. Various longitudinal reports proposed a link between the cortex and subcortex involvement and cognition using quantitative measurement of Aß burden. Neuroimaging studies revealed that Aß deposition in both cortical and subcortical structures was more closely associated with cognitive decline than Aß in the cortex alone [21, 22]. However, to the best of our knowledge, no studies have been performed using visual assessment to investigate the extent of Aß deposition and cognitive progression within the focal Aß involvement.

Our final major finding was that in patients with focal Aß deposition, those who had PPC, striatal, and frontal involvement showed rapid cognitive decline compared to the No-FMM group. These results are further strengthened by previously mentioned studies, which concluded that the subcortical involvement of Aß, in particular striatal Aß, was linked to poorer cognitive performance and more rapid cognitive impairment [21, 23]. Also, our result is in line with another study that detected a regionally specific association between declining memory function and increasing Aß deposition [5]. In that study, this effect was most observed in the posterior cingulate, which is often established as one of the earliest sites of Aß deposition [5]. This opens the possibility for future studies to assess the mechanism of the regionally specific effect of Aß on cognition. These regional effects could be mediated by tau pathology or disrupted functional connectivity [24, 25]. These studies showed that the AD-specific spatial pattern of tau retention in the PPC is a crucial connectivity hub in the cerebral region, which potentially plays an integral part in the development of dementia in AD [24]. In addition, Aβ accumulation on PPC could have an association with hypo-connectivity within the default mode network (DMN) and between the DMN and the front-parietal network [25].

## Limitations

The current study has some limitations. First, we did not have information on the pathological burden of Aß in Focal-FMM patients. Second, the 2-year follow-up period was relatively short. Although the changes in CDR-SB were not large, there were significant differences in the rate of cognitive decline between the groups. Third, we defined “regional Focal-FMM” group as subjects that have ^18^F-flutemetamol uptake in that region irrespective of other regions. Thus, our result might not be true regional specific effect. Further studies are needed to evaluate whether subjects with ^18^F-flutemetamol uptake exclusively in the PPC, striatal, or frontal region show more rapid cognitive decline compared to focal uptake in other regions. Finally, other AD biomarkers such as paired helical filament tau or neurodegenerative markers were not measured. Future research incorporating these markers in the near future may help explain the mechanism underlying effects of focal Aß deposition on cognitive decline.

## Conclusions

In the present study, we identified the cognitive trajectories of patients with focal Aß uptake in a significant number of participants in each cognitive level. Our study results may assist clinicians in managing patients with focal Aβ uptake. When Focal Aβ patients are in the aMCI stage, have a larger extent of focal deposition, or have PPC, striatal, or frontal involvement, the clinician may expect the patient to show cognitive decline within the following few years.

## Supplementary Information

**Additional file 1: Table S1.** The number of ^18^F-flutemetamol uptake regions in cognitively unimpaired, amnestic mild cognitive impairment, and Alzheimer's disease dementia at baseline. **Fig. S1.** Scatter plot and regression line showing a significant correlation between the number of ^18^F-flutemetamol uptake regions and global SUVR.

## Data Availability

The datasets used and analyzed during the current study are available from the corresponding author on reasonable request.

## Abbreviations

Aß: ß-Amyloid
PET: Positron emission tomography
CU: Cognitively unimpaired
aMCI: Amnestic mild cognitive impairment
AD: Alzheimer’s disease
ADD: AD dementia
SD: Standard deviation
KBASE-V: Korean Brain Aging Study for the Early Diagnosis and Prediction of AD
PPC: Precuneus/posterior cingulate
FMM: ^18^F-flutemetamol
CERAD-K: The Korean version of the Consortium to Establish a Registry for Alzheimer’s Disease Assessment Packet
SNSB: Seoul Neuropsychological Screening Battery
K-BNT: Korean version-Boston Naming Test
CDR-SB: Clinical Dementia Rating Scale Sum of Boxes
RCFT: Rey-Osterrieth Complex Figure Test
ANOVA: Analysis of variance
