# Supplementary material for: Cognitive trajectories of patients with focal ß-amyloid deposition
Source: Alzheimers Res Ther. 2021 Feb 19;13:48. doi: 10.1186/s13195-021-00787-7 (PMC7896397; doi:10.1186/s13195-021-00787-7)
Supplement: Supplementary file 1 — Additional file 1: Table S1. The number of 18F-flutemetamol uptake regions in cognitively unimpaired, amnestic mild cognitive impairment, and Alzheimer's disease dementia at baseline. Fig. S1. Scatter plot and regression line showing a significant correlation between the number of 18F-flutemetamol uptake regions and global SUVR. [file 13195_2021_787_MOESM1_ESM.docx]

**Table S1.** The number of ^18^F-flutemetamol uptake regions in cognitively unimpaired, amnestic mild cognitive impairment, and Alzheimer's disease dementia at baseline

| **No. of FMM uptake regions** | **CU (n=112)** | **aMCI (n=78)** | **ADD (n=50)** |
| --- | --- | --- | --- |
| **No-FMM uptake** |  |  |  |
| 0 region | 83 (74.1%)^*^ | 34 (43.6%)† | 5 (10.0%)^‡^ |
| **Focal-FMM uptake** |  |  |  |
| 1 region | 10 (8.9%) | 1 (1.3%) | 0 (0.0%) |
| 2 regions | 3 (2.7%) | 3 (3.8%) | 1 (2.0%) |
| 3 regions | 0 (0.0%) | 1 (1.3%) | 2 (4.0%) |
| 4 regions | 4 (3.6%) | 1 (1.3%) | 2 (4.0%) |
| 5 regions | 2 (1.8%) | 0 (0.0%) | 0 (0.0%) |
| 6 regions | 0 (0.0%) | 2 (2.6%) | 3 (6.0%) |
| 7 regions | 0 (0.0%) | 2 (2.6%) | 2 (4.0%) |
| 8 regions | 0 (0.0%) | 1 (1.3%) | 4 (8.0%) |
| 9 regions | 0 (0.0%) | 0 (0.0%) | 1 (2.0%) |
| **Diffuse-FMM uptake** |  |  |  |
| 10 regions | 10 (8.9%)^*^ | 33 (42.3%) | 30 (60.0%)^‡^ |

*p<0.05 after Bonferroni correction between CU and aMCI

†p<0.05 after Bonferroni correction between aMCI and ADD

^‡^p<0.05 after Bonferroni correction between ADD and CU

Abbreviations: FMM = ^18^F-flutemetamol; CU = cognitively unimpaired; aMCI = amnestic mild cognitive impairment; ADD = Alzheimer’s disease dementia

**Figure Legend**

**
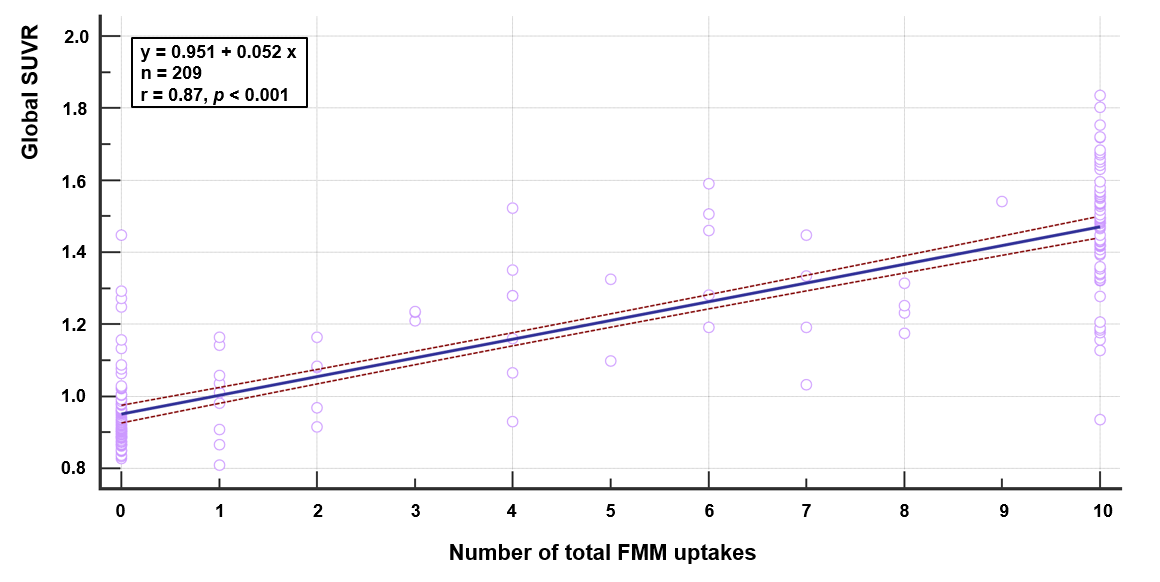
**

**Fig. S1.** Scatter plot and regression line showing a significant correlation between the number of ^18^F-flutemetamol uptake regions and global SUVR.

FMM=^18^F-flutemetamol; SUVR=Standardized Uptake Value Ratio.
